# Variation and Association of Hen Performance and Egg Quality Traits in Individual Early-Laying ISA Brown Hens

**DOI:** 10.3390/ani10091601

**Published:** 2020-09-08

**Authors:** Doreen O. Anene, Yeasmin Akter, Peter C. Thomson, Peter Groves, Cormac J. O’Shea

**Affiliations:** 1 Department of Animal Science, University of Nottingham, Loughborough LE12 5RD, UK; doreen.anene@nottingham.ac.uk; 2 Poultry Research Foundation, University of Sydney, Camden, NSW 2570, Australia; yeasmin.akter@sydney.edu.au (Y.A.); peter.groves@sydney.edu.au (P.G.); 3 School of Life and Environmental Sciences (SOLES), University of Sydney, Camden, NSW 2570, Australia; peter.thomson@sydney.edu.au

**Keywords:** albumen quality, laying hens, feed intake, body weight, egg production

## Abstract

**Simple Summary:**

The feed consumption and feed efficiency, body weight, egg weight, and rate of lay of the hen, as well as the albumen, yolk, and shell characteristics of the egg, are important measures of productivity in the layer industry. While these data are easily collected on a flock basis, there is little information on the extent of variation among individual hens in the literature and much nutrition and management is based on accepting the expected average performance of a cohort of animals. Moreover, body weight or feed consumption may have implications for other traits such as egg or egg component variables. We have attempted to control environmental variables such as feed in order to investigate the inherent variation for these traits present in a flock of 450 individually caged ISA Brown hens in early lay, and their associations with egg measurements. The results from the study revealed important variation in daily feed intake, body weight, and albumen height. Feed intake and hen body weights were weakly associated with egg weight, while feed intake and body weights were not associated with albumen height. Body weight and feed consumption had only a weak linkage with egg weight and did not influence albumen quality.

**Abstract:**

Uniformity in hen and egg traits is an important consideration in commercial layer flocks. There is little information on how individual hen feed consumption and body weight affect egg quality measurements. This study investigated the variation in performance traits of individual hens and associations with egg quality characteristics. Four hundred and fifty-five ISA Brown caged hens in early lay were monitored for 42 days (25 to 30 weeks of age) to collect hen feed consumption and egg production measurements. Forty-four hens from the flock were randomly selected and eggs were collected from the same hen once weekly for albumen, yolk, and shell assessment. The means ± standard deviation of average daily feed intake (ADFI), albumen height, initial body weight (IBW), and final body weight (FBW) were 124 g ± 15, 10.3 mm ± 1.5, 1802 g ± 129, and 2000 g ± 175, respectively. Albumen height was not associated with ADFI (*r* = 0.18, *p* = 0.21), IBW (*r* = −0.04, *p* = 0.79), or FBW (*r* = −0.06, *p* = 0.69). This study showed variation in feed intake, body weight, and albumen quality of individual early-lay hens. Feed intake and body weight did not influence albumen quality.

## 1. Introduction

The average daily feed intake (ADFI), body weight (BW), egg weight (EW), and egg production (EP) of the hen, as well as the albumen, yolk, and shell indices of the egg, are important measures of productivity in the layer industry. Within modern layer strains and production systems, these traits are influenced by many environmental factors such as diet, rearing systems, and disease status [1,2] which lead up to the onset of lay and beyond. In commercial laying flocks, ADFI and EP cannot be measured on an individual hen basis, thus concealing the extent of variation and possible associations linked with egg quality, which may influence decision making towards achieving optimum productivity in the flock. Previous research has explored hen performance traits and/or egg quality on a flock basis [3] or on white-shelled hens [4], however, the extent of variation in performance traits of individual hens and their association with egg quality of the same hens merits investigation [5].

Hen BW is an important contributing factor to egg weight and feed intake [6,7]. Under certain production settings where feed allocation cannot be readily controlled, the BW of ISA Brown hens has been reported to surpass the breed standard recommendation [8]. Excessive body weight can result in lack of laying persistency, production of extremely large eggs causing lower eggshell quality [8,9], and increased fat accumulation causing multiple ovulations and leg health problems [10]. In a study with white-shelled hens ranked by BW [4], it was reported that light BW hens had a higher EP, laid bigger eggs, had a lower feed conversion ratio (FCR), and had higher albumen heights compared to the heavy BW birds. Akter et al. [11] reported associations between traits such as ADFI egg weight and BW, and egg quality characteristics in mid-lay ISA Brown layers. That study showed that hens had varying feed intake but comparable egg mass and hence a low (better) FCR had a higher albumen height. Albumen height is used as an indicator of egg freshness in that it decreases as egg age increases, and also with storage, particularly at higher storage temperatures [12]. The implication of that study was that ADFI and feed efficiency may be associated with indices of egg quality. However, that study was conducted on mid-lay, 55-week-old hens, thus, there are no indications of how early in lay such variation and associations arise, as reports on the variation and associations between hen performance and egg quality are scarce in the literature. 

The aims of this study were to investigate the inherent variation and associations in hen performance traits (feed intake, EP, and BW changes) and egg quality measurements (albumen, yolk, and shell characteristics) of individual ISA Brown hens in an experimental flock of hens during early lay.

## 2. Methodology

The study was conducted at the Poultry Research Foundation, University of Sydney, Australia. All experimental procedures conducted in these studies were approved by the University of Sydney Animal Ethics Committee (AEC number, 2017/1212), and were in accordance with the Australian code for the care and use of animals for scientific purposes (8th Edition, National Health and Medical Research Council, 2013). 

### 2.1. Animal Management and Experimental Design

A total of 455, 21-week-old ISA Brown pullets were weighed individually and randomly allocated to 25 × 50 × 50 cm single metal layer cages. Shed temperature and photoperiod were managed according to the ISA Brown management guide [13]. A wheat/sorghum and soybean meal-based diet formulated to provide 2750 kcal/kg gross metabolizable energy and 16.30% crude protein was offered to the birds on an ad libitum basis in individual feeders. The experimental diet (Appendix A) met the recommended requirements for ISA Brown laying hens in early lay. Hens were provided with water ad libitum via automatic nipples in each individual cage. At the end of the experiment, data from nine hens were excluded from the data set. Three out of the nine hens died prior to the end of the experimental period, while the remaining six had an average EP of 7%.

### 2.2. Production Performance 

At 25 weeks of hen age, data collection of hen performance commenced and lasted for 42 days between 25 and 30 weeks of hen age. This age of hens is important because hen performance and egg quality are known to decline with hen age, thus it is important to determine how early in lay these differences and changes begin. Further, hens averagely start to lay eggs between 18 and 24 weeks and attain peak production at about 25 weeks. The investigation began at 25 weeks to ensure that all hens had attained peak production, but were still in the early stages of lay. Throughout the experimental period, feed consumption, EW, and EP were measured. Individual BW of the hens was taken at 21 (IBW) and 30 (FBW) weeks of age and changes in BW were calculated as the difference between initial and final BW. Average daily feed intake (ADFI) (g) was calculated for individual hens as feed offered minus feed uneaten throughout on a weekly basis and divided by 7, EP (%) was recorded daily per hen throughout the study and was computed weekly as (*n* ÷ 7) × 100, where *n* = number of eggs laid per hen in seven days. Eggs were collected daily and weighed using an electronic scale with a digital output, and egg mass (EM, g) per hen per day was calculated as (EP × EW) ÷ 7, where EP = egg production (number of eggs laid over 7 days) and EW = average egg weight. Feed conversion ratio (FCR) was calculated on a weekly basis as grams of feed consumed per gram of EM for each hen.

### 2.3. Egg Quality Assessment

During the six-week experimental period, a total of 44 hens were randomly subsampled from the flock and eggs were collected once weekly from the same hens for assessment of albumen, yolk, and shell characteristics. Prior to egg breaking, EW (g) was measured using an electronic scale, while egg height (mm) and width (mm) were measured using a 200 mm electronic Vernier caliper (Kincrome, Australia) with a digital readout. Egg shape index was calculated as egg width divided by egg height, multiplied by 100. For internal egg quality testing, eggs were broken out onto a flat, leveled glass surface on a metal stand with a reflective mirror. Before separation, the albumen height, albumen width, and yolk width were measured. Width measurements on the intact egg were taken at the equator, using a Vernier caliper, while albumen height was measured using an albumen height gauge (Technical Services and Supplies, York, UK). Using a plastic scraper, the albumen was separated from the yolk, and yolk height was measured using an AMES tripod micrometer (Waltham, USA). Yolk color was determined using a DSM Yolk Fan (DSM, Switzerland) on a scale from 1 through 16 units. The separated albumen and yolk were weighed individually using an electronic weighing scale. The eggshells were carefully washed, air dried, and weighed with a digital scale. Eggshell membranes were removed, and eggshell thickness was measured at three regions (top, equator, and base) using a caliper. Fresh eggs were collected from the same hens the following day and eggshell breaking strength was measured as the peak force using a texture analyzer (Perten TVT 6700, Stockholm, Sweden) fitted with a cylindrical probe 75 mm in diameter. The Haugh unit was derived using the formula 100 × log_10_ (*h* − 1.7 × *w* ^0.37^ + 7.6), where *h* = albumen height (mm) and *w* = egg weight (g) [3].

### 2.4. Statistical Analysis

Descriptive statistics including means, standard deviation, and coefficient of variation of hen performance and egg quality data were analyzed using SAS^®^ University Edition software. Repeated measures data were analyzed using the MIXED Procedure of SAS to assess between week differences. Pearson or Spearman (for yolk color) correlation coefficients for egg quality measurements were generated using the CORR Procedure in SAS. Differences between weekly means and correlation coefficients values were considered significant if the *p* value was ≤0.05. 

## 3. Results 

### 3.1. Hen Performance 

**Figure 1:**
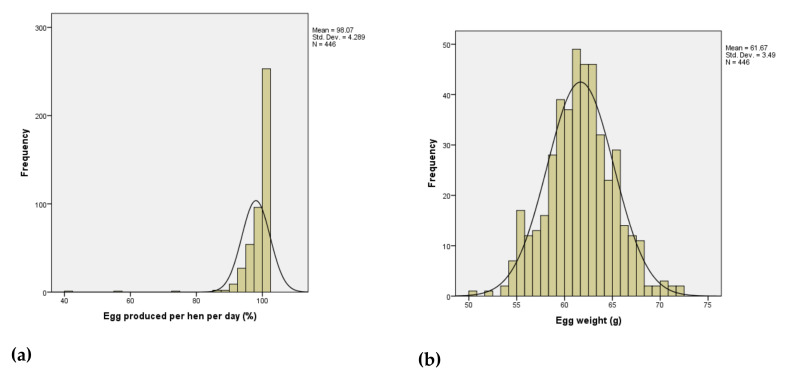

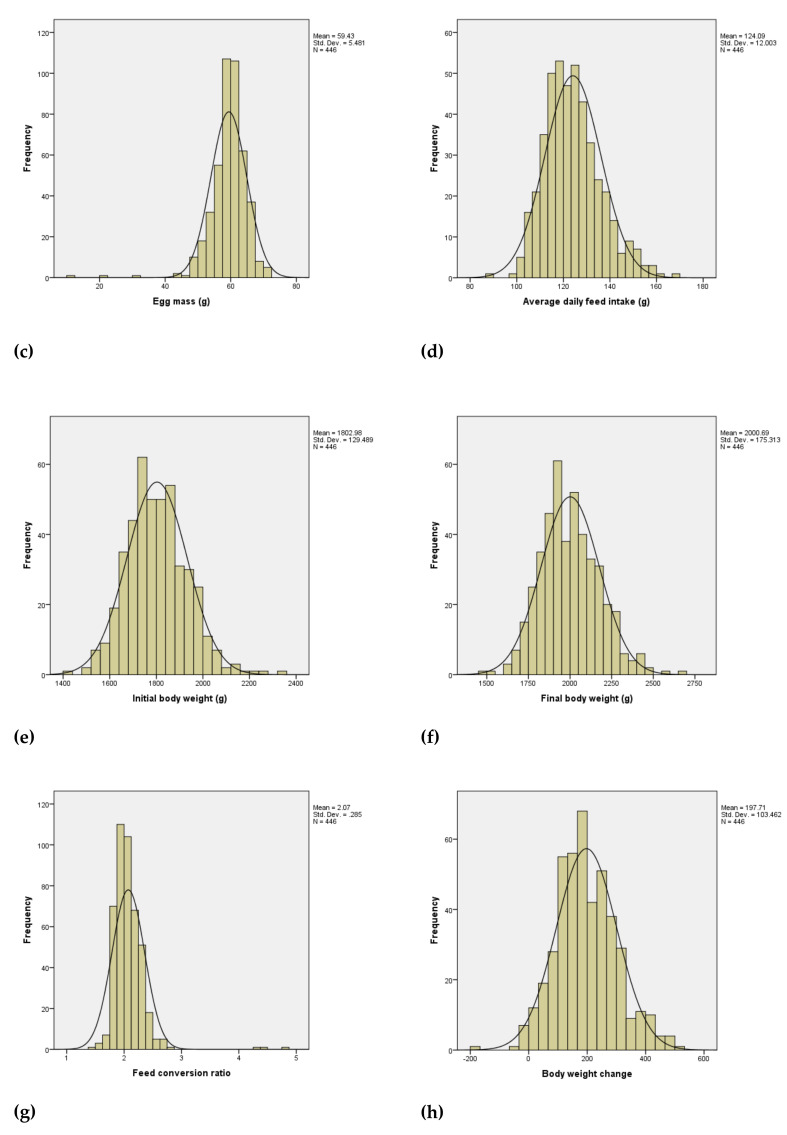
Histogram of performance traits showing distributions of 446 individually caged ISA Brown hens aged between 25 and 30 weeks for (**a**) egg produced per hen per day, (**b**) egg weight, (**c**) egg mass, (**d**) average daily feed intake, (**e**) initial body weight, (**f**) final body weight, (**g**) feed conversion ratio, and (**h**) body weight change.

The summary statistics of individual hen performance between 25 and 30 weeks of age are shown in Table 1 while the histograms of ADFI, EW, and other performance traits are shown in Figure 1.

The hens had an average IBW of 1803 g ± 129 (CV = 7%) at 21 weeks and FBW of 2000 g ± 175 (CV = 9%) at 30 weeks. The ADFI had a CV of 12% while egg weight had a CV of 6%. 

The weekly means of hen performance traits are presented in Table 2. Average daily feed intake increased significantly from 126 g at 25 weeks to 128 g at 30 weeks (*p* < 0.0001). Egg weight also increased significantly from 60 g to 63 g between 25 and 30 weeks of age (*p* < 0.0001). 

The correlations between individual hen performance traits are described in Table 3. The ADFI was positively associated with all BW parameters (*p* < 0.0001) and had a significant but weak positive association with EW (*r* = 0.32; *p* < 0.0001). There was no significant association observed between EP and EW.

### 3.2. Egg Quality

The descriptive statistics of egg quality characteristics are shown in Table 4. Briefly, the mean and standard deviation of albumen height was 10.4 mm ± 1.5, albumen weight was 37.3 g ± 3.0, yolk weight was 17.0 g ± 0.7, and eggshell weight was 6.1 g ± 0.6. The CVs for albumen height, albumen weight, yolk weight, and shell weight were 14%, 8%, 4%, and 10%, respectively. 

Table 5 shows the changes in egg quality indices of individually caged ISA Brown hens between 25 and 30 weeks of age. The height of the albumen decreased from 10.9 mm at 25 weeks of age to 9.1 mm by 30 weeks (*p* < 0.0001), while the width of the albumen increased from 64 mm in the 26th week to 66 mm in the 30th week (*p* < 0.0001).

### 3.3. Hen Performance and Egg Quality Traits

Correlations showing the relationships between hen performance characteristics and egg quality traits of the experimental hens are presented in Table 6. Briefly, there was a weak positive association between ADFI and EW (*r* = 0.32, *p* ≤ 0.001), FBW and EW (*r* = 0.28, *p* ≤ 0.01). ADFI was weakly associated with yolk color (*r* = 0.36, *p* = 0.02) and FCR had a negative association with albumen weight (*r* = −0.49, *p* < 0.0006). There was no association between ADFI and albumen height (*r* = 0.18, *p* = 0.21), IBW and albumen height (*r* = −0.04, *p* = 0.79), or FBW and albumen height (*r* = −0.06, *p* = 0.69).

The linear relationships between some hen performance and egg quality traits are shown in Appendix B. There was a significant relationship between ADFI and BW changes as well as egg weight and albumen height, whereas no relationship was observed between ADFI and albumen quality indices. 

## 4. Discussion 

The monitoring of key hen and egg traits in commercial layer flocks is a critical practice to ensure high levels of productivity and inform on management intervention when necessary. While simple statistics can be collected to assess the extent of variation in flock body weight and egg quality variables, it is not possible to obtain such data on other important variables. According to the ISA Brown breed standard [13], hens aged between 25 and 30 weeks are capable of having an average EP of 96%. The average of 98% recorded in this study confirms that the hens surpassed the expectation of the breed standard, agreeing with the findings of Parkinson et al. [9]. Following the experimental period, nine of the birds were excluded due to mortality (1.5%) or not being in lay (0.4%). For the remainder, the EP of the hens was 98% by week 25 and remained at this level through most of the experimental measuring period with an overall CV of 7%. This shows that once maximum rate of lay is reached, this rate is maintained at least in the reported period of early lay. Hence for most hens in this study, EP was not an important source of variation. 

The weight of the hen egg is an important gauge of preference and quality in the layer industry [14]. The overall average egg weight of 61 g recorded in this study was consistent with the ISA Brown suggested target of 61.5 g for hens between 25 and 30 weeks old, while the egg weight range was between 48 g and 78 g. Together with an average CV of 6%, this suggests that in an early-lay flock, egg weight does not vary considerably. This is corroborated by several studies which reported no significant difference in egg weights from ISA Brown hens [15,16]. However, egg weight increased significantly from week 25 to week 30, at 60 g and 63 g, respectively (*p* < 0.001), and this increase may be due to an increase in hen age, as similarly reported by Lacin et al. [4]. The egg weight recorded at 30 weeks in this study was similar to the results of Lee et al. [17], who reported an average egg weight of 64.1 g ± 3.6 for the HyLine Brown breed of hens at 30 weeks of age. Conversely, the average eggs from the current study were heavier, 61 g, when compared to the work of Roberts et al. [18], who recorded an average egg weight of 57.6 g in their study with ISA Brown hens aged between 25 and 40 weeks old. Taken together, EP and EW were relatively stable over the course of the experimental period, suggesting that these traits were not an important source of variation in the study. 

An ADFI of 115 g per hen per day offered on an ad libitum basis is recommended by the breed standard for hens within 25 to 30 weeks [13]. The ADFI for the total experimental period, 124 g, was higher than the breed expectation of 115 g [13]. The wide range in ADFI, spanning 115 g in the lower quartile of the flock to 169 g in the upper quartile of the flock, together with the 12% average CV in this study, shows dissimilarities in consumption among individual birds and indicates the differences in individual hen appetite and nutritional requirement for performance and maintenance. 

An average FCR of 1.93 is suggested by the ISA Brown breed standard for hens between 25 and 30 weeks. However, the average FCR recorded in this study was 2.10, and this may have been influenced primarily by the high feed intake of the birds in the present study. The calculated weekly FCR ranged from 1 to 3.4 and the 12% average CV demonstrates that considerable differences and variation exist in feed conversion ratio across the flock. There was no effect of time in the FCR between the 25th and 26th week of age, however, weekly FCR was observed to improve (*p* = 0.0008) from 2.17 in the 25th week to 2.13 on the 30th week of age. The higher (worse) FCR observed may be linked to the high feed consumptions rates recorded. 

A total of 70% of the birds weighed more than the suggested range of 1719 to 1900 g [13] for hens between 25 and 30 weeks. This increased BW was similar to observations from Parkinson et al. [8] and Parkinson et al. [9], and is likely to have had implications for the FCR of the hens. Heavier animals have a greater energy and nutrient requirement for maintenance which will increase feed intake, worsen FCR, and subsequently may the affect general health of the hens later in lay [9]. The overall CV for initial and final body weight, which may be used as a measure of uniformity, increased from 7% at 21 weeks to 9% at 30 weeks. This suggests that body weight, although above breed standard, is relatively stable in the early stages of lay in hens offered ad libitum access to feed. The body weight change between initial and final body weight varied markedly in the current study, with a CV of 52%, and this might have implications for greater variation in body weight later in mid- and late lay. The positive association between FCR and BW change in the present study is consistent with a study by Lacin et al. [4], who reported positive associations between BW and FCR. Similarly, Akter et al. [11] reported that hens classed as highly feed efficient, with an FCR value of <1.8, had a significantly lighter BW when compared to hens ranked as poorly feed efficient, FCR > 2.1.

The positive association between hen BW and ADFI in the current study was expected and in agreement with Lacin et al. [4]. The weak associations observed between ADFI and EW and between FBW and EW suggest that increased feed intake and BW had a minor role in the early stages of lay in influencing the weight of the egg. However, as the average BW of hens in this study was in excess of the breed standard, this observation may not apply in flocks with a greater range of BW or a lower average BW. Hence, in the context of this study, the lack of association between BW parameters and EP, and ADFI and EP, suggests that bigger hens did not lay more eggs, and neither did increased feed consumption contribute to more eggs being laid. Various researchers [19,20,21] have suggested that it may be more economical to have lower weight, early-maturing hens which lay consistently compared to heavy hens which lay extra-large eggs. Nordskog and Briggs [22] also investigated the relationship between BW, EW, and EP, and suggested that finding a middle ground to control feed intake and hen body weight while maintaining commercially acceptable egg weights is necessary to maintain uniformity and optimize production resources. Parkinson et al. [9] also highlighted the importance of controlling body weight distribution within the breed standard in order to reduce variation in performance traits among flocks. It may be suggested that, although hen BW plays a significant role in attaining sexual maturity and EP, it may not be as critically important after the maximum rate of ovulation has been reached at about 24 weeks. As observed in the present study, there was no association between EW and BWC. 

Albumen height is an important measure of egg quality and tends to be greatest in early lay and then declines gradually with age [12]. In the present study, the wide range of 7 mm to 14.2 mm and the average CV of 14% for albumen height reflect differences in individual albumen quality of eggs in the early stages of lay. There was a time effect on albumen height which was higher in the 25th and 26th week, compared to the 29th and 30th week. The average albumen height obtained in the present study was similar to that reported by Lee et al. [17]. However, it was higher than the values obtained from the study by Roberts et al. [18] on eggs from a commercial caged layer flock, within 25 to 40 weeks of age. Albumen height can also be corrected for egg weight to determine the Haugh unit [23], which has been adopted by the poultry industry as a standard measure of albumen quality. In the present study, the Haugh unit (HU) of all eggs assessed ranged from 83 to 114, with an average CV of 6.4%, suggesting a generally low level of variability for this egg quality measurement in the flock. There was a significant time effect on the weekly HU (*p* < 0.0001) with a reduction from 103 in the 25th week to 96 in the 30th week. This decline in HU may be associated with the increase in age of the hens also reflected in the decrease in albumen height across the laying period. However, it was surprising to see a time-related decline over such a relatively short experimental period.

The average yolk index recorded in this study was 47%, ranging from 42% to 49%. A higher yolk index implies an increase in quality [24], as the curved shape of the yolk flattens out as the egg ages [25]. The CV of 3% for yolk index from this study suggests uniformity in yolk quality in early-laying hens between 25 and 30 weeks of age. The average yolk index in the current study was higher than that recorded by Mohammadi et al. [26]. Yolk color is an important visual measure of egg quality which is linked to consumer preference [27]. It is influenced primarily by the content and the profile of pigmenting carotenoids present in the feed ingredients [28,29]. The average yolk color score obtained in the current study was similar to that reported in the study by Roberts et al. [18] on hens between 25 and 40 weeks of age. The weak association observed for yolk color and feed intake was expected as yolk color pigment is primarily influenced by dietary fat soluble xanthophylls.

Eggshell characteristics are important measures of quality, determining hatchability, suitability for table, and preference by the consumers [30,31]. The average CVs observed for shell weight and shell thickness suggest uniformity in shell quality across the experimental period. These were similar to those observed in the control group of the study by Mohammadi et al. [26], as well as in the study of early-laying hens reported by Roberts et al. [18]. There was, however, a 15% CV for shell breaking strength. Shell thickness remained relatively consistent throughout the experimental period (*p* = 0.058), with a slight and significant increase of 0.02 mm between the 25th and 30th week of age (*p* = 0.02). There were no associations between hen ADFI, EW, EP, IBW, FBW, and shell thickness and shell breaking strength. This could be linked to the young age of the hens, and it will be useful to see how these relationships continue or change in the later stages of lay. 

In a previous study, it was observed that hens ranked as having a high FCR had greater BW and produced eggs with a lower Haugh unit [11]. In the present study, the absence of an association between feed intake and albumen height, BW indices and albumen height and albumen weight suggests that neither increased feed consumption nor increased hen body weight was associated with albumen quality in early-laying hens. However, as the CV of body weight tended to increase over time (7% at 21 weeks and 9% at 30 weeks) and with the huge variation in BWC, it may be expected that the associations between BW indices and albumen quality could become significant as the hens enter mid-lay, with heavier, inefficient hens producing eggs with lower albumen quality. It would be of interest to investigate the persistence or changes in these associations in the mid- and late lay stages on an individual hen basis.

## 5. Conclusions

This study reports on the extent of variation in EP, EW, ADFI, BW, albumen, yolk, and shell qualities in a flock of individually caged ISA Brown hens in early stages of lay. The body weight of hens in this study was in excess of BW guidelines of ISA Brown. Albumen height and ADFI varied considerably between individual early-laying birds. Hen body weight and feed consumption at the early stages of lay had only a weak association with egg weight, and there were no significant associations between ADFI and albumen height or between body weight and albumen height. It can be concluded that there is important variation among hen performance and egg quality traits, as well as associations between early-laying hens and eggs. Further investigation on the persistence and changes in the variation and its association with egg quality is merited to better understand the links between production traits and egg quality and safety.

## Figures and Tables

**Table 1:** Descriptive statistics of hen performance of 446 individually caged ISA Brown hens aged between 25 and 30 weeks old.

Performance Trait		25 to 30 Weeks				
	*n*	Mean	Min	Max	SD	CV (%)
Eggs produced per hen/week (%)	2676	98	0	100	6.8	7.0
Egg weight (g/day)	2676	61	48	78	3.8	6.2
Egg mass (g/day)	2676	59	0	77	6.3	11
Average daily feed intake (g/day)	2676	124	60	191	15	12
Feed conversion ratio	2676	2.1	1.0	3.4	0.2	12
Initial body weight, 21 weeks (g)	446	1803	1434	2342	129	7.2
Final body weight, 30 weeks (g)	446	2000	1476	2668	175	8.8
Body weight change (g)	446	198	-194	522	103	52

SD: standard deviation; CV: coefficient of variation; Min: minimum; Max: maximum.

**Table 2:** Changes in mean hen performance traits of individually caged ISA Brown hens between 25 and 30 weeks of age (*n* = 2676).

	Age of Hen (Weeks)							
	25	26	27	28	29	30	SEM	*p*-Value
Egg produced per hen per day (%)	98.6 ^c^	98.1 ^ac^	98.3 ^ac^	97.6 ^ab^	97.2 ^b^	98.5 ^c^	0.32	0.012
Egg weight (g)	60 ^b^	61 ^c^	62 ^a^	62 ^a^	62 ^a^	63 ^d^	0.18	<0.0001
Egg mass (g)	58 ^b^	59 ^bc^	60 ^ad^	59 ^ac^	59 ^bc^	60 ^d^	0.29	<0.0001
Average daily feed intake (g)	126 ^ab^	126 ^ab^	125 ^a^	117 ^c^	123 ^d^	128 ^b^	0.70	<0.0001
FCR	2.17 ^a^	2.13 ^ab^	2.08 ^c^	1.98 ^d^	2.08 ^e^	2.13 ^bc^	0.01	<0.0001

SEM: standard error of mean; ^a,b,c,d^ means with different superscripts are significantly different (*p* < 0.05).

**Table 3:** Correlation (*r*) among hen performance traits of early-laying ISA Brown hens aged between 25 and 30 weeks old; *n* = 446 hens.

	EP	EW	ADFI	IBW	FBW	BWC	FCR
EP	1						
EW	−0.03	1					
ADFI	0.07	0.32 ***	1				
IBW (21 weeks)	−0.10	0.36 ***	0.50 ***	1			
FBW (30 weeks)	0.01	0.28 ***	0.63 ***	0.81 ***	1		
BWC	0.15 *	0.03	0.45 ***	0.12	0.68 ***	1	
FCR	−0.66 ***	−0.15 *	0.5 ***	0.27 ***	0.32 ***	0.2 **	1

*** coefficients with a *p* value < 0.0001; ** coefficients with a *p* value < 0.01; * coefficients with a *p* value of ≤0.05; BWC: body weight change; EW: egg weight; IBW: initial body weight; FBW: final body weight; ADFI: average daily feed intake; EP: egg produced per hen/day; FCR: feed conversion ratio.

**Table 4:** Descriptive statistics of egg quality indices of 44 individually caged ISA Brown hens aged between 25 and 30 weeks old (*n* = 264).

Egg Quality Trait	Mean	Min	Max	SD	CV (%)
Egg height (mm)	55	51	60	1.7	3.1
Egg width (mm)	44	42	57	1.3	2.9
Egg shape index (%)	79	71	100	2.9	3.7
Albumen height (mm)	10	7.0	14	1.5	14
Albumen width (mm)	64	55	82	3.7	5.8
Albumen weight (g)	37	31	46	3.0	8.1
Albumen index (%)	16	11	26	2.7	17
Albumen percentage (%)	61	55	76	2.3	3.8
Haugh unit	100	83	114	6.4	6.4
Yolk weight (g)	17	15	19	0.7	4.0
Yolk width (mm)	37	34	42	1.3	3.6
Yolk height (mm)	14	10	18	1.2	8.5
Yolk percentage (%)	23	18	52	2.5	11
Yolk index (%)	47	42	49	1.5	3.4
Albumen: yolk ratio	2.6	2.1	3.7	0.3	10
Yolk color	11	11	13	0.5	4.3
Shell weight (g)	6.1	3.7	7.6	0.6	10
Shell thickness (mm)	0.4	0.3	0.4	0.02	4.4
Shell percentage (%)	10	6.7	12	0.8	8.6
Shell breaking strength (g)	4852	3129	6952	731	15

CV: coefficient of variation; Min: minimum; Max: maximum; egg shape index = egg width/egg length × 100; albumen percentage = albumen weight/egg weight × 100; albumen index = albumen height/albumen width × 100; yolk percentage = yolk weight/egg weight × 100; yolk index = yolk height/yolk width × 100; albumen: yolk ratio = albumen weight/yolk weight; shell percentage = shell weight/egg weight × 100.

**Table 5:** Changes in egg quality indices of individually caged ISA Brown hens between 25 and 30 weeks of age (*n* = 264).

	Age of Hen (Weeks)							
	25	26	27	28	29	30	SEM	*p*-Value
Albumen height (mm)	11 ^ab^	11 ^a^	10.5 ^abc^	10.4 ^bc^	10 ^cd^	9.3 ^d^	0.21	<0.0001
Albumen width (mm)	64 ^a^	62 ^c^	64 ^a^	64 ^a^	65 ^ab^	66 ^b^	0.53	<0.0001
Haugh unit	103 ^a^	103.3 ^ab^	101 ^abc^	100.5 ^bc^	98.6 ^c^	96 ^d^	0.88	<0.0001
Yolk height (g)	13.1 ^a^	13.2 ^a^	13.9 ^b^	14.3 ^bc^	14.6 ^c^	14.2 ^bc^	0.16	<0.0001
Shell breaking strength (g)	4886 ^a^	5043 ^a^	4839 ^a^	4806 ^ab^	5004 ^a^	4533 ^a^	107.1	0.0151
Shell thickness (mm)	0.40 ^ab^	0.40 ^a^	0.41 ^abc^	0.42 ^bc^	0.42 ^abc^	0.42 ^c^	0.002	0.058

^a,b,c,d^ means with different superscripts are significantly different (*p* < 0.05).

**Table 6:** Correlation (*r*) between hen performance and egg quality parameters of individually caged ISA Brown hens aged between 25 and 30 weeks old (*n* = 44).

	Egg Shape Index	Albumen Height	Albumen Weight	Yolk Weight	Yolk Height	Yolk Color	Albumen: Yolk	Shell Weight	Shell Thickness	Eggshell Breaking Strength
Egg produced/hen/day	−0.12	0.08	−0.13	0.17	0.04	0.04	−0.12	−0.16	0.06	0.001
Egg weight	0.08	0.31 *	0.91 ***	0.33 *	0.38 **	−0.11	0.54 **	0.73 ***	−0.13	−0.004
Egg mass	−0.01	0.32 *	0.69 ***	0.44 **	0.33 *	−0.08	0.39 **	0.51 **	−0.07	−0.003
Feed intake	−0.03	0.18	−0.04	0.15	0.23	0.36 *	−0.18	0.08	0.09	0.03
Initial body weight	−0.07	−0.04	0.09	−0.12	0.14	0.11	−0.001	−0.08	−0.005	−0.07
Final body weight	0.01	−0.06	0.03	0.03	0.15	0.20	−0.05	0.001	0.10	0.007
Body weight change	0.09	−0.05	−0.05	0.19	0.09	0.23	−0.08	−0.09	0.17	0.09
Feed conversion ratio	−0.05	−0.03	−0.49 **	−0.08	0.01	0.38 *	−0.43 *	−0.28	0.12	0.02

*** coefficients with a *p* value < 0.0001; ** coefficients with a *p* value < 0.01; * coefficients with a *p* value of ≤0.05.

## Appendix A

Hen diet used in study, formulated by a commercial nutritionist who creates the same diets for poultry farmers.

**Table A1:** Dietary composition of the experimental basal diet.

Feed Ingredient	Amount (kg/Tonne)
Wheat 10.5%	346.80
Sorghum 12%	345.00
Soybean meal 46%	155.00
Limestone grit 38%	71.00
Canola expel 32%	30.00
Limestone	20.00
Dicalcium phosphate	15.00
Soybean oil	7.00
Sodium bicarbonate	3.19
DL-methionine	1.75
Lysine-HCl	1.70
Salt	1.60
Layer premix *	1.00
L-Threonine	0.45
Choline chloride 60%	0.30
Ronozyme WX CT (DCM)	0.12
Ronozyme Hi-phosphate layer 300 (DCM)	0.03
Total	1000.00
Calculated Nutrient Analysis	
Crude protein (%)	16.30
Gross metabolizable energy (kcal/kg)	2750.00
Net energy (kcal/kg)	2106.00
Crude fat (%)	2.90
Total digestible lysine (%)	0.74
Total digestible methionine (%)	0.39
Total digestible tryptophan (%)	0.18
Total digestible isoleucine (%)	0.58
Total digestible arginine (%)	0.83
Total digestible valine (%)	0.65
Calcium (%)	4.00
Linoleic acid (%)	1.39
Crude ash (%)	13.40
Sodium	0.18
Lysine (%)	0.82
Total Phosphorus (*p*) (%)	0.61
Available (*p*)	0.40
Methionine (%)	0.42
Xanthophyll (mg/kg)	6.00

* each kilogram contained vitamin A 15 000 IU; cholecalciferol 1 500 ICU; dl-α-tocopheryl acetate 30 IU; menadione 5.0 mg; thiamine 3.0 mg; riboflavin 6.0 mg; niacin 20.0 mg; pantothenic acid 8.0 mg; pyridoxine 5.0 mg; folic acid 1.0 mg; vitamin B1 15 μg; M 80.0 mg; Z 60.0 mg; F 30.0 mg; Cu 5.0 mg; 2.0 mg; and Se 0.15 mg.

## Appendix B

**Table A2:** Linear regression between hen performance and egg quality indices (*n* = 44).

Outcome Variable	Predictor Variable	Slope ± SE	Intercept ± SE	*R* ^2^	*p*-Value
Feed intake	Albumen height	1.76 ± 1.41	104.32 ± 14.73	0.036	0.22
Feed intake	Egg weight	0.143 ± 0.55	113.8 ± 34.25	0.002	0.79
Body weight change	Albumen height	−3.99 ± 11.43	236.66 ± 119	0.003	0.73
Body weight change	Egg weight	1.306 ± 4.44	115.34 ± 272.3	0.002	0.77
Egg weight	Albumen height	0.78 ± 0.37	53.07 ± 3.94	0.093	0.04
EP	Albumen height	0.147 ± 0.252	96.72 ± 2.63	0.008	0.56
EP	Body weight	0.004 ± 0.003	97.45 ± 0.72	0.33	0.24

EP—Egg produced per hen per day (%).
